# The Royal Frederick's Hospital, Copenhagen

**Published:** 1892-10-29

**Authors:** 


### HOSPITAL ADMINISTRATION.

### THE ROYAL FREDERICK'S HOSPITAL, COPENHAGEN.

The official report for last year's working of this celebrated Hospital, with the attached "Royal Birth and Foster Institutions," contains various items of interest. The number of patients directly brought to the hospital was 2,403 (1,207 men, 1,196 women), against 2,623 for the previous year. Of these patients 1,644 attended themselves to their conveyance to the hospital, whereas 759 had to be fetched by the hospital's carriages. Of the patients, 1,906 belonged to Copenhagen, 470 came from other parts of Denmark, and 27 from abroad. The patients injured by accident amounted to 292. Of the patients, 887 paid for their treatment at the hospital—58 of these availed themselves of the reduction granted to several societies, &c., and only paid 80 öre (about 10d.) per diem; 1,516 patients were received free of any charge. The aggregate number of treatment days was 114,605, of which 29,856 days come upon paying and 84,749 days upon non-paying patients. The annual expenditure per patient amounted to 1,110 kr. against 1,079 kr. the previous year. The highest number of patients was on January 28th, viz., 358 persons. The smallest number was 210. Of the patients 2,145 have left the hospital, 248 have died, 78 have been transferred to other sections. The proportion of mortality was was 0·1,036 against 0·1,173 the previous year. Close upon 7,000 baths of various kinds have been served, of which some 4,700 baths have been given to non-patients free of any charge. The hospital's number of beds was 377. Residences for a larger number of intending sick nurses (pupils) have been erected. A new laboratory has also been arranged.

To the "Royal Birth Institution" were during the year conveyed 1,537 women, of whom 27 left the institution without giving birth to any child. Under the auspices of the institution, but in apartments or residences chosen by themselves, 22 women gave birth, 5 died, and 58 were transferred to various hospitals; 664 women gave birth at the institution itself, and 838 at its branch establishments. One woman gave birth to triplets and 16 to twins. Of the women in question 284 were married, the remaining 1,216 unmarried. Fifty-eight women, of whom only 2 were married, paid for their stay and treatment at the institution. Of 1,535 children, 72 were stillborn and 56 died shortly after the birth; 938 children were taken away without, or at least provisionally without, foster help; 469 were transferred to the Foster Home. It was noted as a remarkable instance that out of 1,500 women confined not one single woman died from fever—a thing that would have been thought impossible thirty years ago. During the year 35 midwives left the institution, having passed the requisite examination.

